# A virtual reality classroom to teach and explore crystal solid state structures

**DOI:** 10.1007/s11042-022-13410-0

**Published:** 2022-08-11

**Authors:** Erica Stella, Isabella Agosti, Nicoletta Di Blas, Marco Finazzi, Pier Luca Lanzi, Daniele Loiacono

**Affiliations:** Politecnico di Milano, Milan, Italy

**Keywords:** Virtual reality, Education, Virtual classroom, Solid state physics, Crystallography

## Abstract

We present an educational application of virtual reality that we created to help students gain an in-depth understanding of the internal structure of crystals and related key concepts. Teachers can use it to give lectures to small groups (10-15) of students in a shared virtual environment, both remotely (with teacher and students in different locations) and locally (while sharing the same physical space). Lectures can be recorded, stored in an online repository, and shared with students who can either review a recorded lecture in the same virtual environment or can use the application for self-studying by exploring a large collection of available crystal structures. We validated our application with human subjects receiving positive feedback.

**Supplementary Information:**

The online version contains supplementary material available at 10.1007/s11042-022-13410-0https://doi.org/10.1007/s11042-022-13410-0.

## Introduction

**Fig. 1:**
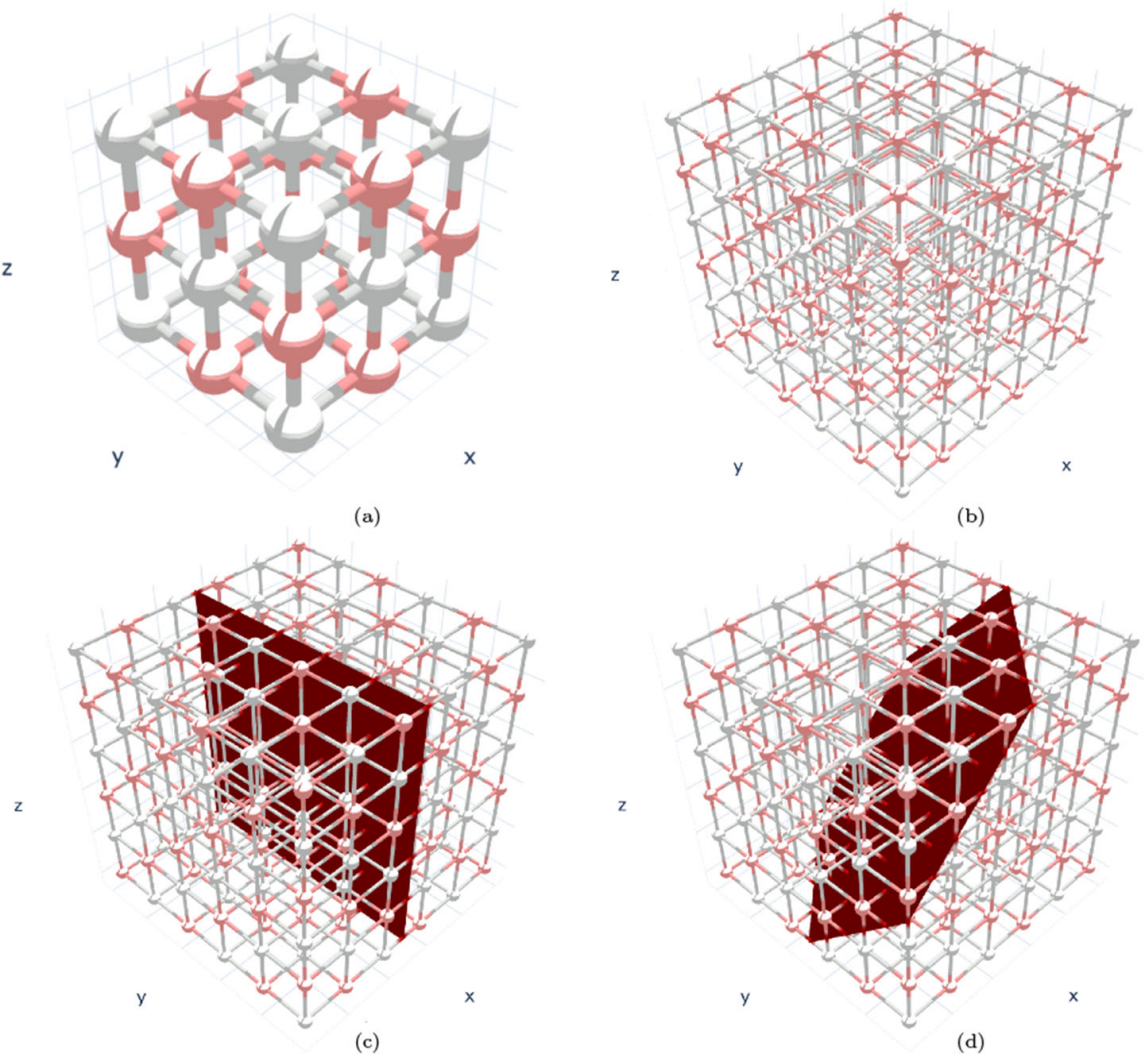
Sodium Chloride (NaCl) (a) unit cell; (b) unit cells on the crystal lattice; (c) the plane corresponding to the 100 Miller index; and (d) the plane corresponding to the 111 Miller index. Images have been created using nanoHUB Crystal Viewer [16]

Crystals are ubiquitous in nature and represent a key element in several scientific fields such as mineralogy, chemistry, physics, metallurgy, geology, biology, and medicine [8]. A crystal is defined by its unit cell and its lattice geometry. The former specifies the arrangement of atoms in the material and represents the minimum crystal size possible. The latter describes how the unit cells are orderly arranged in three dimensions to make up the crystal structure. Unit cells lie in layers or planes that are identified by their *Miller indices*, a triplet of integer values (*hkl*) that determine the vector normal to the plane. Figure 1 shows, for the simple sodium chloride crystal (NaCl), (a) its unit cell, (b) unit cells positioned on the lattice geometry, the planes corresponding to (c) Miller index 100 (the one having x is the vector normal to the plane), and (d) Miller index (111).

Crystals form complex three-dimensional structures that can be difficult to visualize using traditional two-dimensional representations like images in books or flat displays. Accordingly, students usually find it difficult to understand the spatial arrangement of the atoms and to master how some key concepts, like Miller indices, map on complex crystal structures. Physical artifacts (made out of plastic, metal or wood) are more effective than two-dimensional images; however, they are bulky to store, time-consuming to build and modify. There are several commercial and open source molecular visualizers (e.g., Crystal Viewer [16]) and some of them support virtual reality head sets (e.g., [18]). Academia has also been very active in this area with studies focused on applying computer graphics to achieve more accurate depictions of the chemical compounds and their properties while also reducing the rendering speed [44]. Several applications also deal with teaching crystals-related concepts to students [9]. However, most visualizers are single-user and the ones that have multi-user support focus on collaborative tasks rather than teaching [14, 31].

In this paper, we present an educational application of virtual reality we created to help students understand crystal structures either (i) by attending online lectures that take place in a shared virtual environment or (ii) by exploring a library of crystals structures. Immersive virtual environments have been shown to help students grasp more clearly the 3D shapes of objects [62, 63]. Existing tools mainly focus on self study (e.g. [9]) and collaborative problem solving (e.g., [14, 31]). In contrast, our application has been designed specifically as a support for online university lectures with a focus on the student-teacher class interaction and after-class activities. Teachers can use it (i) to give online lectures to small groups (10-15) of students in a shared virtual environment, and (ii) to record lectures that students can later watch in the same virtual environment. Students can use it as a self-study tool that let them explore a library of crystal structures and recorded lectures. We designed the application in collaboration with professors who, over the last decade, have been teaching crystallography lectures for the“Solid State Physics” course at Politecnico di Milano. Our colleagues helped us identify the functionalities useful to teachers and students during online lectures, for recording a lecture, and for after-class activities. They provided us with an extensive list of actions that users should be able to perform when interacting with crystal structures. Finally, since cost and ease of use are essential factors, they asked us to support inexpensive and user-friendly devices. To the best of our knowledge, this is the first educational crystallography application to provide support for online lectures in a virtual environment to small student classes for low-cost stand-alone devices.

We evaluated our application with 30 human subjects during two events, one involving engineering students, one involving people attending a public scientific dissemination event. The evaluation focused on usability, the effectiveness in conveying the spatial arrangement of atoms inside crystal structures, the fundamental concepts of crystal structures (like Miller indices), and a qualitative assessment of issues related to motion sickness. Subjects received a brief introduction about how the experience would unfold and how to use the controller. Next, they wore the headset and attended a brief virtual reality lecture covering fundamental crystallography concepts and the available functionalities. After the lecture, subjects were allowed to explore the library of crystals on their own. The experience lasted for about 15 minutes and, at the end, we asked subjects to complete an anonymous online form with 27 questions asking for (i) basic background information; (ii) feedback regarding usability, comfort, and educational value of the application using Likert scale [35] or True/False values; and (iii) general suggestions and comments using open questions. Overall, we received very positive feedback suggesting that our application can be an effective mean to convey the spatial arrangement of atoms inside crystals and student would enjoy and benefit from having it as a support to crystallography lectures.

The paper is organised as follows. In Section 2, we provide an overview of the recent research on molecular visualisers and virtual reality applications for teaching crystals-related topics. We describe the application in detail in Section 3 and its high-level software architecture in Section 4. In Section 5, we present the results of an evaluation we carried out both with engineering students and general public. Finally, in Section 6, we draw some conclusions and outline future works.

## Related work

Virtual reality enables users to experience situations that would either be impossible in real life (like doing a space walk [55]), too dangerous (like training as a firefighter [11, 12, 70]), or too expensive [75]. Several studies have shown that skills gained in virtual simulations may be successfully transferred to real life situations [42, 53] and retained over a longer period of time [10]. Accordingly, virtual reality has been applied to training and learning in a broad spectrum of domains including industrial processes and security [38, 40], aviation safety [10], cultural heritage [3, 4], military training [54, 69], Internet of Things [77] surgery [1, 59, 65, 71], geography [39], safety procedures [10], and social interactions [36, 37, 64, 72, 73]. Virtual reality has also been widely applied to education and a thorough survey of the research in such broad area is outside the scope of this paper. Accordingly, we refer the interested reader to the recent surveys for a more complete overview of this area [21, 24, 28, 52, 61].

In this section, we present a brief overview of the most relevant works on the visualization and teaching of chemical structures published over the last ten years. Table 1 summarizes the works we selected organized by type of structure and purpose. Research in this area focuses either (i) on biology, thus on the visualization and simulation of protein structures (Table 1, column Biology) or (ii) on the visualization of crystal structures (Table 1, column Crystallography). In the former case, data are represented using the Protein Data Bank (PDB) format [6], in the latter, data are represented using the Crystallographic Information File (CIF) [7]. The goal is usually the scientifically accurate visualization of such complex structures, the simulation of interactions, or the support for teaching (see the rows in Table 1).

**Table 1:** Works on the visualization and teaching of chemical structures published over the last ten years, organized by type of structure and purpose, that were selected for our overview

	Biology	Crystallography
Visualization	– Screens [32, 43, 48, 60]	– Screens [16, 47, 58]
	– Virtual Reality [29, 45, 49, 57]	– Virtual Reality [18, 50]
	– Augmented Reality [44, 76]	– Augmented Reality [41, 66, 67]
Simulation	[14, 17, 19, 30, 31, 33, 46]	[22, 23]
Teaching	[5, 13, 15]	[9, 51, 68]

### Biology - visualization

Interactive visualization of molecular structures dates back to a time when computer did not exist [20] and, as such, it represents one of the oldest branches of data visualization. Kozlíková et al. [32] presents an extensive overview of representation models, rendering, visualization, and simulation for structural biology. The review discusses the major technical challenges while it does not consider virtual reality nor augmented reality. Most of the visualization tools in this area focus on scientific accuracy and typically support flat displays like for example, PyMOL [60], cellVIEW [43], and Chimera [48]. Some tools support virtual reality headsets like the recent versions of VMD [29] and ChimeraX [49], the evolution of Chimera [48]. Molecular Rift [45] integrates virtual reality with Microsoft Kinect V2^1^ to enable hand-based interactions; Caffeine [57] uses a more expensive CAVE-based approach. Fewer tools exploit augmented reality like for example ChemPreview [76], based on Meta 1^2^, and [44] based on Microsoft Hololens^3^ to reproduce proteins. Interestingly, in both cases, the authors reported being constrained in several circumstances by the platforms hardware limitations.

### Biology - simulation

Some tools integrates accurate visualization with scientific simulations of molecular interactions; accordingly, they might also be included in the previous category. Férey et al. [19] designed a virtual reality framework dedicated to immersive and interactive molecular simulations that also integrates haptic devices for manipulation. Other tools let users collaborate. For example, Lee et al. [33] built a collaborative system for the visualisation of biomolecular structures to overcome the spatial and temporal limitations of different workplaces. It employed back-projection displays and polarised glasses for stereoptic viewing. Connor et al. [14] designed a virtual reality framework for molecular dynamics that allowed local cooperation among users in the same room. These would wear a visor tethered to a computer while simulations and synchronization happened on a remote server. Kingsley et al. [31] created a collaborative virtual reality application for the visualization of macromolecular structures of drugs. Their goals is the democratization of drug discovery tools to enable people with different backgrounds join in and quicken the field. O’Connor et al. [46] developed a multi-user virtual reality framework, called Narupa, that enables groups of researchers to cohabit a real-time simulation environment to manipulate the dynamics of complex molecular structures. Deeks et al. [17] applied Narupa to test different protocols for docking to the SARS-CoV-2 Main Protease. Juarez-Jimenez et al. [30] present a framework combining molecular dynamics simulation with virtual reality to explore large amplitude conformational changes in protein structures.

### Biology - teaching

All the systems discussed so far can also be used for teaching and therefore could be broadly considered also in this category. However, only few tools have been designed specifically for teaching right from the start. Coan et al. [13] created two virtual reality laboratories (one focused on DNA and collagen, the other one on hemoglobin). Students access one of the labs and initially read a short powerpoint presentation; next they are presented with short tasks (e.g. determine the dimensions of the three DNA molecules using a measuring tool). At the end they receive a questionnaire about the learned concepts. Similarly, Bennie et al. [5] designed a real-time interactive molecular dynamics simulations in virtual reality that included a set of three short tasks that students had to complete (e.g., rearrangement, unbinding, and docking in specific scenarios). Dai et al. [15] developed a virtual reality framework with body tracking to help students better understand coordination chemistry and molecular orbitals. All these tools implement single-user experiences and reported positive feedback from students although none of them presented the data nor the results of an experimental evaluation like we do in this paper.

### Crystallography - visualization

There are fewer tools for the visualization of crystals than the ones available for biomolecules. Furthermore, most of them use flat displays like RasMol [58] and OpenRasMol [47], Crystal Viewer [16], Jmol^4^, and [25]. Indeed, some tools support virtual reality. Drouhard et al. [18] proposed a visualiser for materials science using the Oculus Rift^5^ head mounted display. VRChem is another virtual reality tool to visualize complex material science molecules developed for a master thesis project [50]. Other tools exploits augmented reality [41, 66, 67]. For example, Swamy et al. [66, 67] and Mansoor [41] developed two applications to visualise stereoisomers and crystal structures. The former relied on mobile phones and was based on Vuforia^6^; the latter used the more expensive Microsoft Hololens. Both explained their choice to use augmented reality as a way to reduce the cognitive load required by imagining complex spatial structures in order to ease the learning process. More recently, Zakharov et al. [74] discussed the potential benefits of using virtual and mixed reality in studying the geometry of the crystal lattice; however, the authors did not implement any tool.

### Crystallography - simulation

NOMAD VR [22, 23] is probably the most important project in this category and it comprises applications running on a wide variety of virtual reality platforms, from CAVE, to several models of head-mounted displays, to mobile-based ones like Google Cardboard^7^. It is an open source project for the visualization and the simulation of interactions between molecules and materials with a long list of features, whose availability varies based on the hardware available.

### Crystallography - teaching

As noted before, all the visualization and simulation tools might be used to support class activities. As such, all the crystallography tools considered so far could be included in this category. There are however few tools that have been specifically developed for teaching. Quishpe-Armas et al. [51] designed virtual laboratory for tethered headsets that a student can use to learn the 3D spatial arrangements of crystals that are necessary to micro-electro-mechanical systems (MEMS). Tarng et al. [68] designed a virtual reality material science teaching module aimed at instructing students on shape memory alloys. Caro et al. [9] did a pilot study to examine whether virtual reality could improve the performances of students with respect to a traditional paper-based lesson. Accordingly, they built a virtual reality learning module about crystal structures aimed at students of a basic material science and engineering course. In [9], they report the results of a four questions form that was presented to 7 students (4 using the virtual reality tool, 3 using the paper-based learning). Although, the sample size did not allow them to draw general conclusions, the authors noted that students using the application answered better when spatial reasoning was required. Interestingly however, students who used the paper-based module had higher scores when questions needed remembering prior knowledge. Note that, these tools for teaching crystallography were mainly designed as single-user experience focused on a set of learning activities to be completed [51, 68]. In contrast our framework was designed as a virtual reality class experience with the focus both on (i) a live teacher-student multi-user interaction and (ii) offline single-user experience involving the viewing of recorded lectures and the free exploration of the teaching material.

## The virtual reality crystallography application

We created the application as a tool to support the studying and teaching of crystal structures. Teachers can use it as a virtual classroom or a recording studio to produce study material that students can access in the same virtual environment. Students can improve their understanding of crystal structures by attending live online lectures in an interactive virtual environment or by exploring an interactive library of crystals structures and recorded lectures.

### Requirements

At first, we extensively interviewed our colleagues that have taught crystallography at Politecnico di Milano over the last ten years. They demonstrated the tools they have been using and highlighted their limitations. They asked us to design an application (i) that would provide accurate three-dimensional visualization and manipulation of crystals; (ii) that instructors could use for teaching; (iii) students could use to deepen their understanding and review study material. Our colleagues specified a list of interactions (e.g., move, zoom, and rotate, apply symmetries and cuts) and asked to have the option to record both the live lectures with students and after-class material. Being cost an essential factor for a broad adoption of the application, we were asked to support inexpensive devices.

We discussed various options including augmented reality using smartphones, Microsoft Hololens, and virtual reality using the inexpensive and stand-alone head mounted display Oculus Go. At the end, we agreed to use virtual reality because of several limitations of augmented reality. Firstly, augmented reality using smartphones would still display the crystals on a flat surface; in contrast, virtual reality enables a realistic three-dimensional view of the crystals. Secondly, virtual reality provides a potentially infinite world that can be used to place the crystals; while with augmented reality, crystals might overlap with real objects (e.g., furniture) and accurate positioning would require high-end devices equipped with Lidar^8^ cameras. Furthermore, virtual reality headsets isolate from the outside world, thus improving focus and limiting environmental distractions. Finally, even though we decided to use Oculus Go, one of cheapest virtual reality head-mounted displays on the market, it is way cheaper and has more computational power when compared to current augmented reality devices.

### Preliminary evaluation

We carried out a preliminary evaluation of an initial prototype with our colleagues teaching crystallography. The qualitative evaluation mainly centered around (i) the scientific accuracy of the educational content, and (ii) the usability of the interface. We focused on our colleagues since they would have been in charge of introducing the application to students during their courses and showcasing it. Thus, it was essential for us that they would approve its educational content, find it comfortable, and consider it a valid support for their curricular activities. We planned to include students in the next and more thorough evaluation (see Section 5). Given the qualitative nature of the evaluation, we did not hand out a questionnaire but simply recorded the opinions and suggestions. The evaluation highlighted some minor issues regarding the zooming and panning of crystals which could overlap with the interface, which we fixed. It also gave teachers the opportunity to ask for additional features like for instance, (i) the option to visualize crystals without bounds, using uniform colors, with the size of atoms proportional to their atomic weight; (ii) a section devoted to epitaxial crystals [2]. All the features were included in the final version.

### Access modes

The application can be accessed as a teacher to organize online lectures (in teacher-student mode) and to record material that students can watch later (using the recording mode); or as a student to access recorded material, and to explore the library of available crystals (in student-mode).

#### Teacher-student mode

**Fig. 2:**
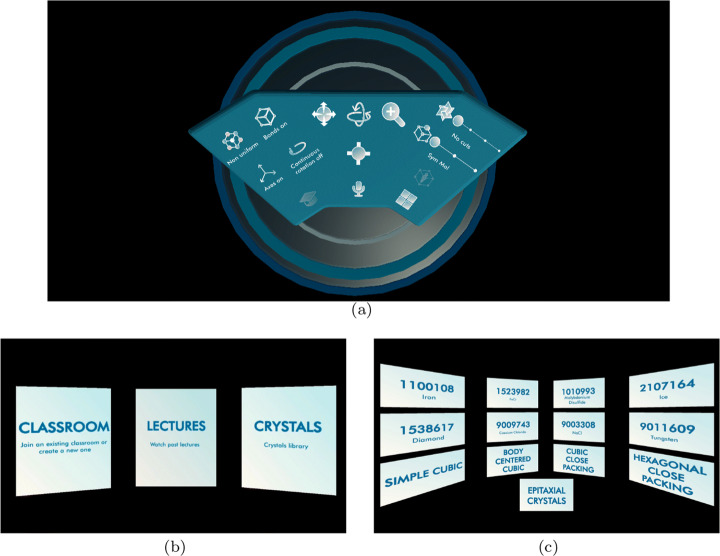
Screenshots from the application main user interfaces: (a) the virtual desktop; (b) mode selection; (d) the library menu

Teachers are the only users that can create a virtual classroom and start a lesson. They start it as a shared environment with a unique identifier and timestamp that students can join by selecting it from a list of the available classes. Teachers can check the list of connected participants so that, once all the students are present, the lesson can begin. Note that an authentication is requested upon accessing the application to identify the users’ role (teacher or student). During a lecture, teachers are the only ones allowed to interact with the structures using all the functionalities made available on their virtual desk shown in Fig. 2a. Students are spectators and can only watch the manipulations applied to the crystals while listening to the explanation. Virtual classes can be organized remotely, with students and teacher in different locations, or locally, with everybody staying in the same room. When teaching remotely, the headset microphone is active and captures the teacher voice that is sent to the speakers embedded in the headsets of the other participants.

#### Recording mode

Teachers can record lectures (i) online, while teaching to a class or (ii) offline, while preparing material for a lecture. In both cases, the interactions with the crystals are done in the same way through the desk (Fig. 2a), but all the manipulations applied on the crystal and the recorded voice memos are saved on an online server. After lectures are uploaded to the server, they receive a unique identifier including the date and time of the recording; students can then access and replay the recorded lectures at any time.

#### Student mode

Students can use the application to watch available recordings or to explore the library of crystal structures by themselves. In order to apply the different manipulations, students use the same desk (Fig. 2a) professors use in the Teacher-Student Mode.

### Interface

We evaluated several interface models during a series of preliminary sessions with teachers. At the end, based on their feedback, we decided to use a desk metaphor (Fig. 2a). The desk is positioned over a circular platform surrounded by void and everything users can do is activated by a widget on the desk—either a button, a switch, or a discrete slider. Teachers and students found this setup more familiar and made them feel more at ease in the virtual environment. The circular platform and the fixed desk channel users towards the desk, implicitly cutting off all other possible moves (which would be impracticable anyway).

The desk is flat to provide the widest field of view over the crystal structures (initially, we experimented with vertical transparent panels but they occluded the view too much). In addition, the interactive elements are positioned to minimize the need for moving the head and grouped based on the type of interaction. Commands for switching between views of the crystals (like, the molecule, the unit cell, the lattice, and Miller index planes) are positioned on the right side of the desk. Commands used more often (like panning, rotating, and scaling) are placed in the center. Commands for modifying the overall view (like, switching the bonds on and off, or using a uniform color and representation of atoms) are position on the left side of the desk.

**Fig. 3:**
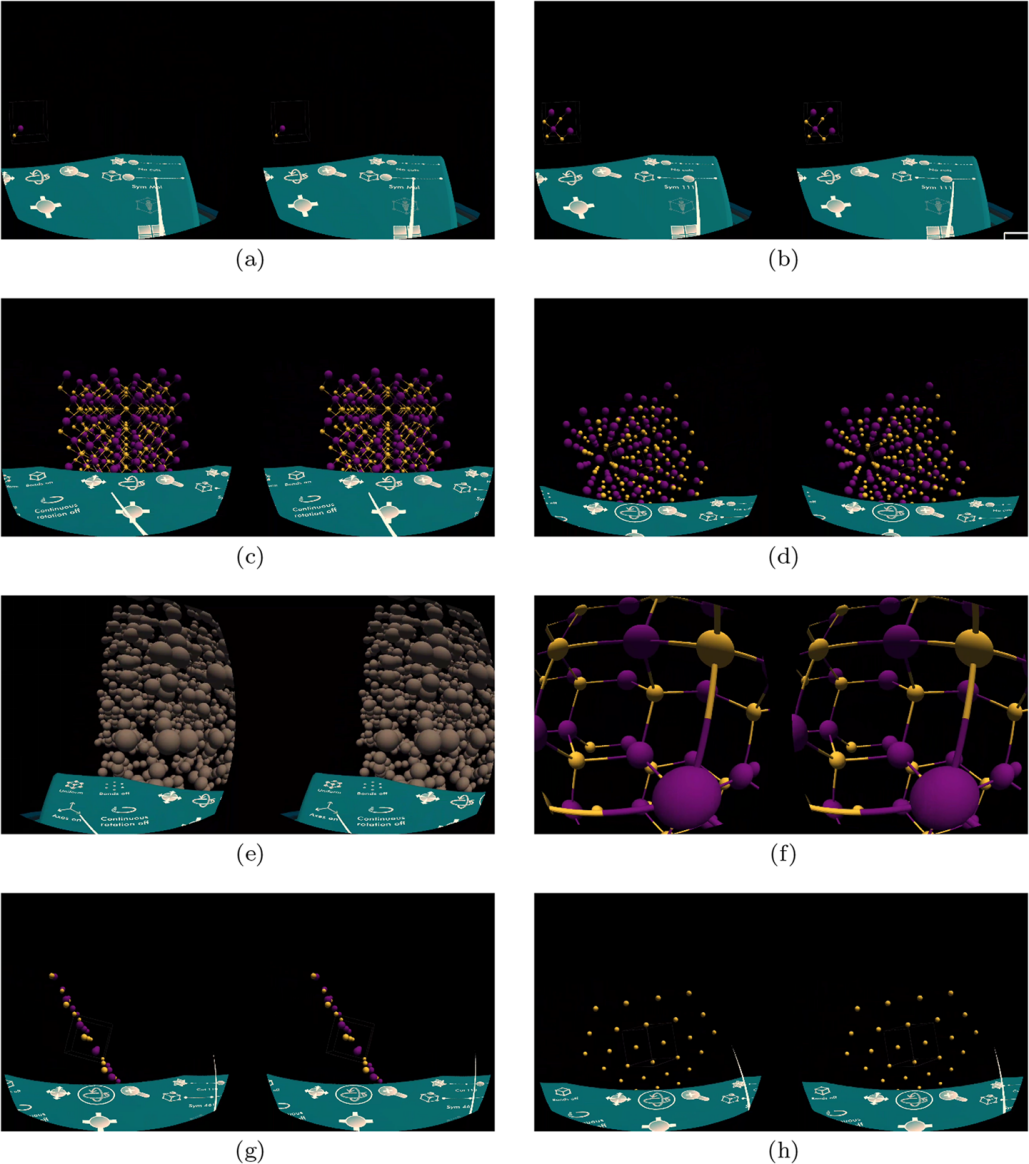
Screenshots from the application learning experience: (a) the diamond molecule; (b) its unit cell; (c) the lattice with unit cells; (d) the lattice without bonds; (e) using the uniform view; (f) inside the crystal; (g) Miller index 110; (h) Miller index 111

Figures 2 and 3 show images taken from a session. As the application starts, users can choose to access an existing class, the recorded lectures, or the library (Fig. 2b). When the library is selected, users are taken to the menu of available structures (Fig. 2c) that are identified by a unique id (e.g., 1538617 for diamonds and 1100118 for iron). The list of available crystals was specified by our colleagues. Crystals are stored using the Crystallographic Information File (CIF) [26, 27], a standard text file format developed by the International Union of Crystallography (IUCr).^9^ The Crystallography Open Database^10^ provides access to thousands of structures in CIF format that can be potentially included in our application, making it a general-purpose tool for crystallography. In the example, when selecting the diamond structure, users are positioned at the desk and the simplest building block of a diamond is shown (Fig. 3a); they can visualize its unit cell (Fig. 3b) and the unit cells on the diamond lattice geometry (Fig. 3c). Crystals can be rotated and the visualization of the bonds (the tubes connecting the atoms) can be switched off (Fig. 3d). Structures can also be visualized using uniform colors and with the size of atoms proportional to their atomic weight (Fig. 3e). Users can get an inside view of the crystal (Fig. 3f); in this case users can look around but cannot move or rotate the crystal since this would cause vection which would result in motion sickness. With a button of the controller the inside crystal view is turned off and the users is returned at the desk. This transition is implemented by the typical fade off/fade on transition used in virtual reality teletransportation to avoid vection or motion sickness. Finally, users can examine planes corresponding to different Miller indexes (Figs. 3g and 3h).

## Architecture

The application was designed for stand-alone and inexpensive head mounted displays. Both the initial prototypes and the first version of the applications were developed for Oculus Go^11^.

### Oculus go

was the first low-cost stand-alone virtual reality head-mounted display (HMD). It has a single 5.5-inch LCD display with a resolution of 1280×1440 pixels per eye and a refresh rate of 72 or 60 Hz, depending on the application. Its lenses provide a field of view of about 101 degrees. Input is provided with a wireless controller that behaves like a laser pointer. Oculus Go provides a non-positional 3-degrees-of-freedom tracking, making it capable of seated or static-standing activities.

### Software architecture

**Fig. 4:**
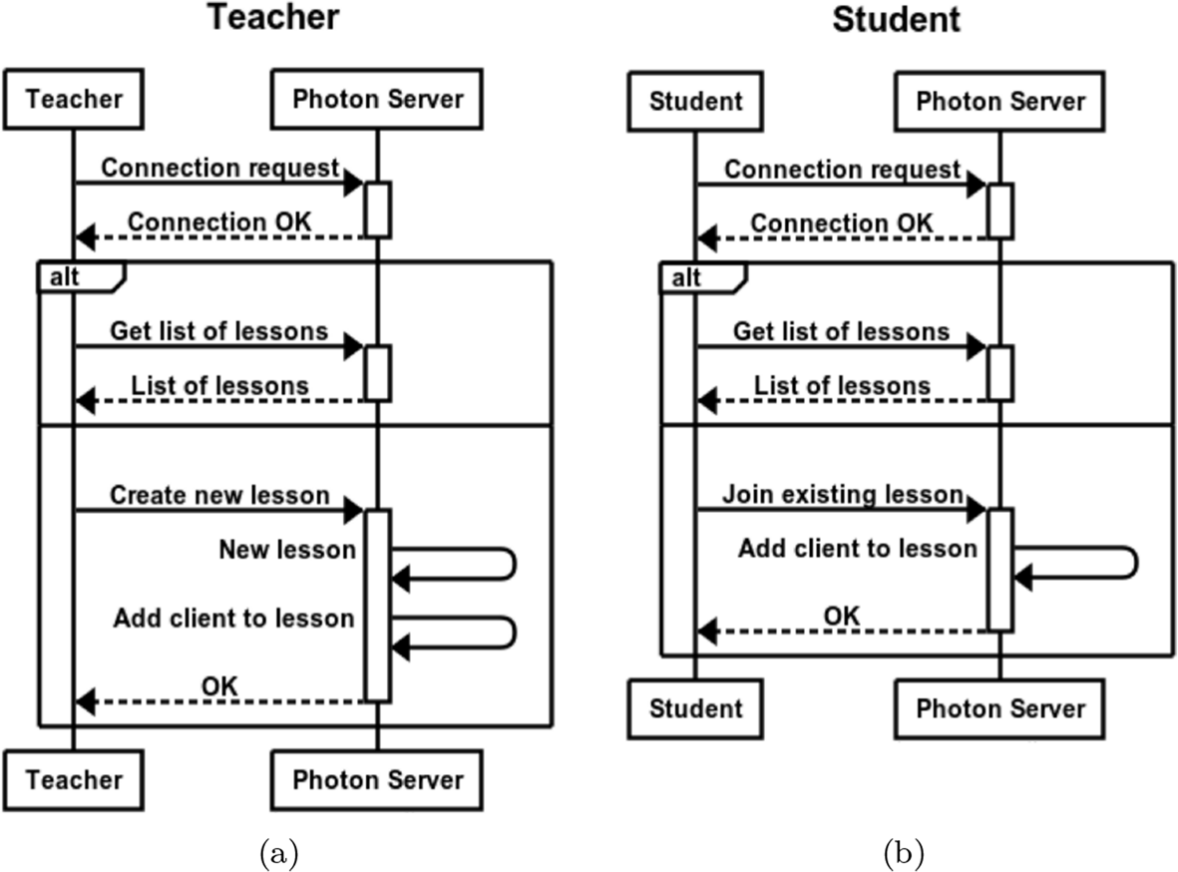
The interaction workflows for teachers and students to connect to the application server

**Fig. 5:**
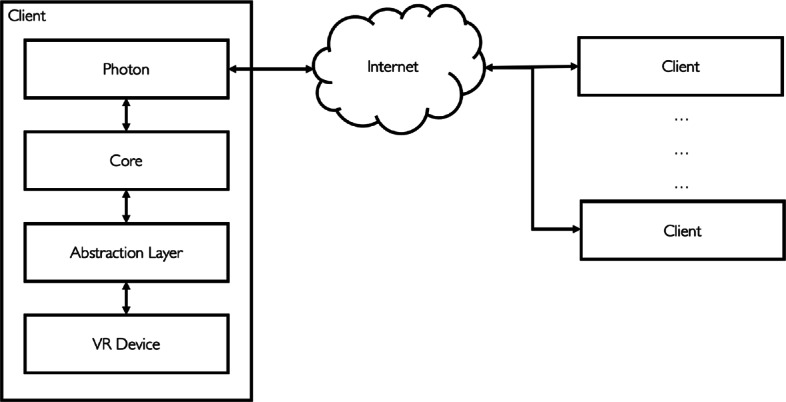
The software architecture

The application was developed using Unity^12^ and the Photon Engine^13^ for online multi-user support. Photon offers dedicated servers, authentication integration, matchmaking and in-game communication. It also provides executables that users can use to run their own server locally. The application relies on a client-server architecture consisting of a single server and multiple clients. Teachers connect to a lobby where they can create a new lecture and invite students to join. During the lecture, all the teacher actions and comments are sent to the server that forwards the information to the students’ clients in real-time. Figure 4 shows the interaction workflows for teachers and students to start and access a virtual lecture. At first, clients connect to a server hosted on the Photon Cloud or on a local server. Next, clients can request the list of all the available lessons, create a new one (if they assigned a teaching role) or join an existing one. Servers keep lectures separated so that clients can interact only with other clients connected to the same lesson. Note that, Recording Mode does not involve any real-time communication since students can access the available lectures later, at any time, by accessing the platform in Student Mode, which also does not involve any real-time interaction with other users. Figure 5 shows the high-level software architecture of our application. The *Core* implements the platform-independent functionalities such as the creation, visualization and manipulation of crystals, access to the libraries models. Because of the relative large number of headset models and the rapid evolution of the technology, we encapsulated the interaction with our application in an abstraction layer that facilitates porting it to new devices. The layer maps the available functionalities to the actual platform used. Thus, we can potentially support multiple platforms, with different interaction patterns and input devices, by writing platform-specific modules that manage the input and call the functionalities exposed by the *Abstraction Layer*. Note that, we implemented our own abstraction layer since other solutions like Unity XR Interaction Toolkit^14^ is still in preview.

## Experimental evaluation

The evaluation was carried out during two different events, one involved engineering students, one was carried out during a public scientific dissemination event.

### Setup of experiments

**Fig. 6:**
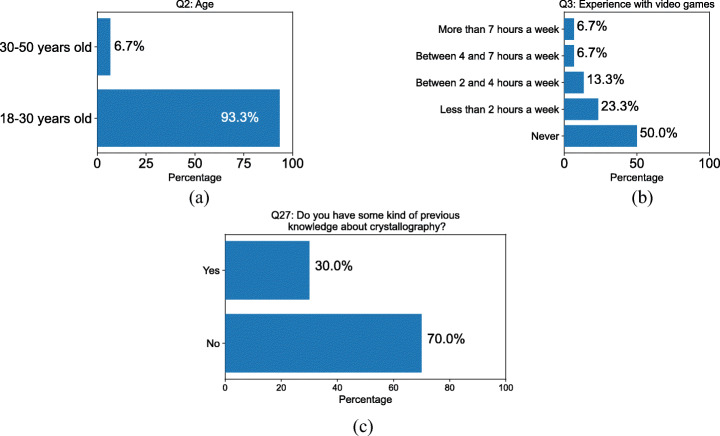
Answers to questions about age (Q2), previous experience with video games (Q3), and crystallography (Q27)

We gave subjects a brief introduction to the experience, described its purpose, how it would unfold, and how to use the controller. Next, they wore the headset and started the application as students attending a brief crystallography lecture (that is, in Teacher-Student mode) focused on the fundamental crystallography concepts. During the lecture the subjects were introduced to the available functionalities. At the end of the lecture, subjects were allowed to try explore the library of available crystals on their own (in Student Mode). Overall the experience lasted for about 15 minutes and, at the end, subjects were asked to complete an online form to evaluate their experience. The form included 27 Likert-scale [35] questions reported in Table 2, which extends the IBM Usability Questionnaire [34]. It was specified that, when answers ranged from 1 to 5, 1 meant “I completely disagree” or “Very poor” and 5 meant “I completely agree” or “Very good”. For all the questions, we report the percentage of answers in each category as well as the overall mean and standard deviation values. We had 30 subjects (21 males and 9 females), mostly between 18 and 30 years old (only two were between 30 and 50 years old), and 9 of them had previous knowledge about crystallography (Fig. 6a). 15 subjects did not play video games; 7 played video games less than 2 hours per week; 4 played from 2 to 4 hours per week; 2 played from 4 to 7 hours per week; 2 subjects played for more than 7 hours per week (Fig. 6b); 9 subjects had some previous knowledge about crystallography, the remaining 21 had none (Fig. 6c).

**Table 2:** Questionnaire filled out by users to evaluate their experience

Id	Question	Answer Type
Q1	Gender	Male/Female/Other
Q2	Age	One out of:
		∙ Less than 18 y/o
		∙ Between 18 and 30 y/o
		∙ Between 30 and 50 y/o
		∙ More than 50 y/o
Q3	Experience with using video games	One out of:
		∙ Not at all
		∙ Less than 2 hours per week
		∙ 2 to 4 hours per week
		∙ 4 to 7 hours per week
		∙ More than 7 hours per week
Q4	Moving in the virtual environment was easy	Likert Scale 1-5
Q5	Activating commands in the virtual environment was easy	Likert Scale 1-5
Q6	I felt comfortable in the virtual environment	Likert Scale 1-5
Q7	I understood within a reasonable time what I could do in the virtual environment	Likert Scale 1-5
Q8	Please add any comment/suggestion you deem useful	Open
Q9	How would you rate the usability of the hand-held device	Likert Scale 1-5
Q10	How would you rate the usability of the 3D environment	Likert Scale 1-5
Q11	How would you rate the usability of the elements within the environment that can be activated/deactivated	Likert Scale 1-5
Q12	How would you rate the usability of the overall experience?	Likert Scale 1-5
Q13	Please add any comment/suggestion you deem useful	Open
Q14	Did you feel comfortable during the overall experience?	Likert Scale 1-5
Q15	I always felt comfortable during the overall experience	True/false
Q16	I felt comfortable at the beginning, but after some minutes I did not feel well	True/False
Q17	I did not feel comfortable at the beginning, but after some minutes I did well	True/False
Q18	Please add any comment/suggestion you deem useful. In particular, we would like to know what annoyed you the most (if anything)	Open
Q19	How easy was it to understand the meaning of the panels on the desk?	Likert Scale 1-5
Q20	How easy was it to understand the meaning of the buttons on the desk?	Likert Scale 1-5
Q21	How easy was it to understand the connection between commands and the different representations of the crystal?	Likert Scale 1-5
Q22	Please add any comment/suggestion you deem useful. In particular, we would like to know what was MOST UNCLEAR to you	Open
Q23	In your opinion, could this kind of experience enrich a traditional university or high-school lecture?	Likert Scale 1-5
Q24	Would you appreciate lessons with this kind of support?	Likert Scale 1-5
Q25	Overall, did you enjoy the experience?	Likert Scale 1-5
Q26	Please add any comment/suggestion you deem useful	Open
Q27	Do you have some kind of previous knowledge about crystallography?	Yes/No

### Experimental results

The questionnaire asked subjects to evaluate the experience in terms of usability, comfort, interface, and educational value.

#### Usability

**Fig. 7:**
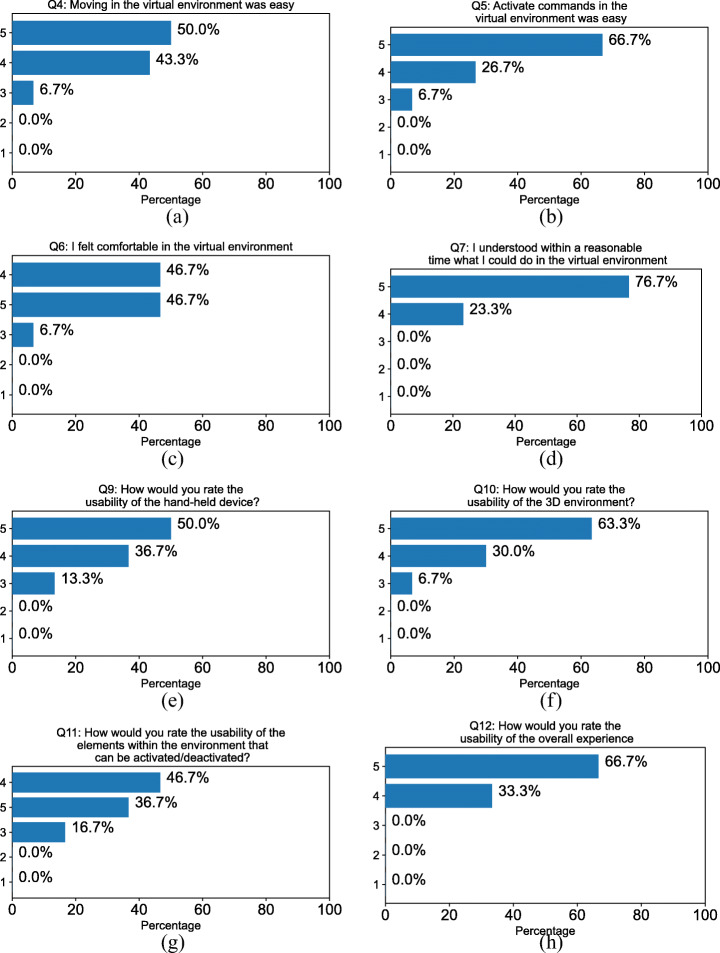
Answers to questions about the usability (Q4-Q12) of the experience

The subjects rated the usage of the virtual environment as cozy and intuitive (Fig. 7). Moving in the virtual environment was easy (Fig. 7a Q4 4.43 ± 0.62) as well as using the various functionalities (Fig. 7b Q5 4.6 ± 0.61). Subjects felt comfortable in the virtual environment (Fig. 7c Q6 4.4 ± 0.61) and could understand within (what they perceived as) a reasonable time what they could do in the environment (Fig. 7d Q7 4.77 ± 0.42). Subjects rated very high both the usability of the hand-held device (Fig. 7e Q9 4.37 ± 0.71), of the environment (Fig. 7f Q10 4.57 ± 0.62), and the usability of the elements within the environment that can be activated/deactivated (Fig. 7g Q11 4.2 ± 0.70). Their evaluation of the experience, as a whole, was very good (Fig. 7h Q12 4.67 ± 0.47). In the comments section of Q8, two subjects stated they initially had problems interacting with the application because they did not know how to use the controller. However, after some additional training, the problems were solved.

#### Comfort

**Fig. 8:**
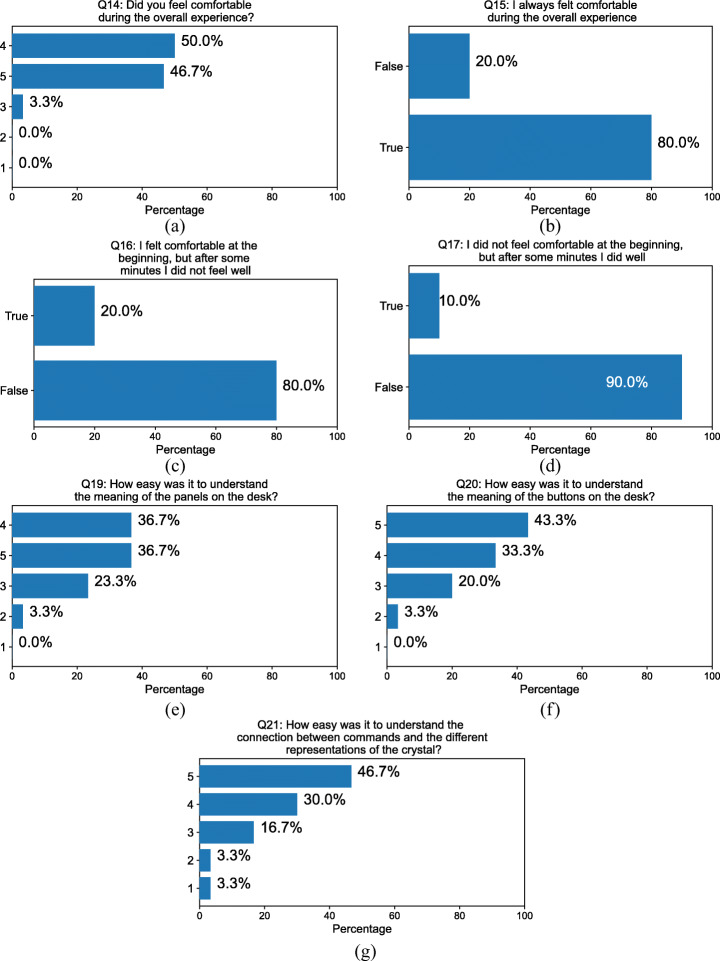
Answers to questions about comfort (Q14-Q17) and interface intuitiveness (Q19-Q21) of the experience

Most of the subjects felt comfortable during the whole experience (Fig. 8a Q14 4.43 ± 0.56), 20% of the subjects felt uncomfortable during part of the experience (Fig. 8b Q15 80% True). Some subjects reported some dizziness during the experience: 20% felt comfortable initially but later did not feel well (Fig. 8c 20% True); 10% felt initially uncomfortable but later in the experience did well (Fig. 8d 20% True). The answers to Q18 showed that dizziness was mainly experienced after seeing crystals rotating during the lecture (in Teacher-Student mode) when subjects did not have control over the environment. Interestingly, two subjects experienced some dizziness when looking down and not seeing their body. Other two wrote that the headset was not very comfortable to wear and easily went out of focus. This is a common issue with headsets that do not allow to change the interpupillary distance, like the Oculus Go, since people that vary significantly from the average head size tend to suffer the misalignment with the lenses.

#### Interface

In terms of intuitiveness, subjects reported that the meaning of the panels and the buttons on the desk was easy to understand (Fig. 8e Q19 4.07 ± 0.85 and Fig. 8f Q20 4.17 ± 0.86) as well as it was the effect these had on the displayed crystal (Fig. 8g Q21 4.13 ± 1.02). The high value of standard deviation in Q21 was mainly due to people who had no prior knowledge in crystallography and had problems in understanding what effect symmetries and cuts had on the visualization.

#### Educational value

**Fig. 9:**
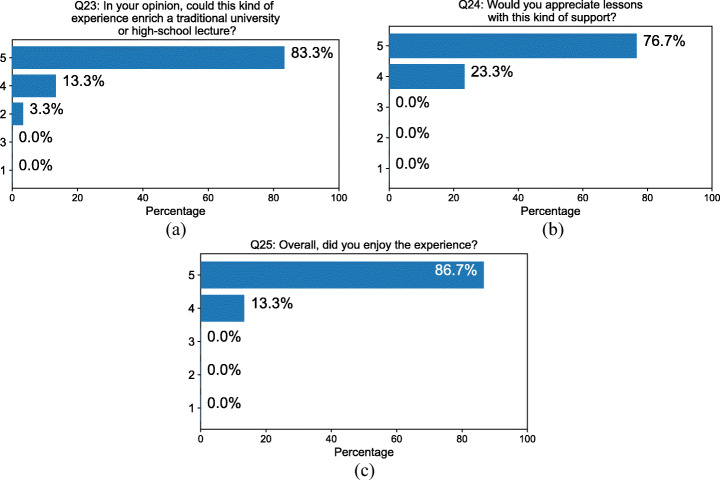
Answers to questions about the educational value (Q23-Q25) of the experience

Almost all the subjects believed that the experience could enrich traditional lectures at high-school and university level (Fig. 9a Q23 4.77 ± 0.63) and stated that they would appreciate class activities with this type of support (Fig. 9b Q24 4.77 ± 0.43). Overall, they greatly enjoyed the experience (Q25 4.87 ± 0.35). In the open question box, From the open questions we received some interesting suggestions: two subjects proposed to add the name of the crystal on the desk, so that people do not forget which crystal they chose; some suggested to add a fake body showing legs and feet when looking down; one suggested to lower the lighting. Table 3 summarizes all the results of the evaluation while Table 4 reports the raw data collected from the questionnaires.

**Table 3:** Summary of the results from the evaluation questionnaires. 0% values are not reported as they correspond to answers that no subject selected

Question	Percentage of answers	*μ* ± *σ*
Q1	Male (70.0%); Female (30.0%)	–
Q2	Between 18 and 30 y/o (93.3%)	–
	Between 30 and 50 y/o (6.7%)	–
Q3	Not at all (50.0%)	
	Less than 2 hours per week (23.3%)	
	2 to 4 hours per week (13.3%)	
	4 to 7 hours per week (6.7%)	
	More than 7 hours per week (6.7%)	–
Q4	5 (50.0%); 4 (43.3%); 3 (6.7%)	4.43 ± 0.62
Q5	5 (66.7%); 4 (26.7%); 3 (6.7%)	4.60 ± 0.61
Q6	5 (46.7%); 4 (46.7%); 3 (6.7%)	4.40 ± 0.61
Q7	5 (76.7%); 4 (23.3%)	4.77 ± 0.42
Q9	5 (50.0%); 4 (36.7%); 3 (13.3%)	4.37 ± 0.71
Q10	5 (63.3%); 4 (30.0%); 3 (6.7%)	4.57 ± 0.62
Q11	4 (46.7%); 5 (36.7%); 3 (16.7%)	4.20 ± 0.70
Q12	5 (66.7%); 4 (33.3%)	4.67 ± 0.47
Q14	4 (50.0%); 5 (46.7%); 3 (3.3%)	4.43 ± 0.56
Q15	True (80.0%); False (20.0%)	–
Q16	False (80.0%); True (20.0%)	–
Q17	False (90.0%); True (10.0%)	–
Q19	5 (36.7%); 4 (36.7%); 3 (23.3%); 2 (3.3%)	4.07 ± 0.85
Q20	5 (43.3%); 4 (33.3%); 3 (20.0%); 2 (3.3%)	4.17 ± 0.86
Q21	5 (46.7%); 4 (30.0%); 3 (16.7%); 2 (3.3%); 1 (3.3%)	4.13 ± 1.02
Q23	5 (83.3%); 4 (13.3%); 2 (3.3%)	4.77 ± 0.62
Q24	5 (76.7%); 4 (23.3%)	4.77 ± 0.42
Q25	5 (86.7%); 4 (13.3%)	4.87 ± 0.34
Q27	No (70.0%); Yes (30.0%)	–

**Table 4:** Raw data from collected questionnaires

Question	Number of Answers Per Available Option
Q1	Male (21); Female (9)
Q2	Between 18 and 30 y/o (28)
	Between 30 and 50 y/o (2)
	Not at all (15)
	Less than 2 hours per week (7)
Q3	2 to 4 hours per week (4)
	4 to 7 hours per week (2)
	More than 7 hours per week (2)
Q4	5 (15); 4 (13); 3 (2)
Q5	5 (20); 4 (8); 3 (2)
Q6	5 (14); 4 (14); 3 (2)
Q7	5 (23); 4 (7)
Q9	5 (15); 4 (11); 3 (4)
Q10	5 (19); 4 (9); 3 (2)
Q11	4 (14); 5 (11); 3 (5)
Q12	5 (20); 4 (10)
Q14	4 (15); 5 (14); 3 (1)
Q15	True (24); False (6)
Q16	False (24); True (6)
Q17	False (27); True (3)
Q19	5 (11); 4 (11); 3 (7); 2 (1)
Q20	5 (13); 4 (10); 3 (6); 2 (1)
Q21	5 (14); 4 (9); 3 (5); 2 (1); 1 (1)
Q23	5 (25); 4 (4); 2 (1)
Q24	5 (23); 4 (7)
Q25	5 (26); 4 (4)
Q27	No (21); Yes (9)

## Conclusions and future work

We presented an educational application we designed to help teachers and students involved in crystallography classes at Politecnico di Milano. Teachers can use it to organize online lectures in a shared virtual environment, and to record material that students can later watch in the same virtual environment. Students can use it to attend online virtual classes, to watch recording in the same virtual environment, or to explore a library of available crystals. While existing applications focus on the visualization of complex crystal structures, our application was designed *specifically* as a support for online university lectures, with a focus on the student-teacher class interaction and after-class activities. We evaluated the application with engineering students and people attending a scientific dissemination event, receiving positive feedback both in term of usability, comfort, and engagement.

As future developments, we plan to introduce the possibility for students to interact with the professor by raising a virtual hand and being able to take control of the presentation desk (similarly to what is done in many video conferencing tools). The development tool and the networking middleware we used (Unity and Photon) already support these functionalities. However, the feature has not been implemented yet since teachers feared it might be too difficult to manage in virtual reality. As teachers will gain more experience in online virtual classrooms, we are confident that they might be more willing to interact with students more dynamically. We also plan to introduce a gamified mode with quizzes and simple tasks, that might help students’ self-assessment on specific topics. Finally, we have ported the application to the Oculus Quest^15^ headset family and we are evaluating the introduction of controller-free interactions, similar to what analyzed in [56], that Oculus Quest models now support.

## Electronic supplementary material

Below is the link to the electronic supplementary material.

(MP4 118 MB)

## Footnotes

1 https://en.wikipedia.org/wiki/Kinect

2 https://en.wikipedia.org/wiki/Meta_(company)

3 https://en.wikipedia.org/wiki/Microsoft_HoloLens

4 http://jmol.sourceforge.net

5 https://www.oculus.com

6 https://www.ptc.com/en/products/augmented-reality/vuforia

7 https://arvr.google.com/cardboard/

8 https://en.wikipedia.org/wiki/Lidar

9 https://www.iucr.org/

10 http://www.crystallography.net/cod/

11 https://www.oculus.com/go/

12 http://www.unity.com

13 https://www.photonengine.com/en/

14 https://docs.unity3d.com/Manual/XR.html

15 https://www.oculus.com/quest/
